# Rietveld refinement of a natural cobaltian mansfieldite from synchrotron data

**DOI:** 10.1107/S1600536808044127

**Published:** 2009-01-10

**Authors:** Matteo Zoppi, Giovanni Pratesi

**Affiliations:** a Museo di Storia Naturale – Sezione di Mineralogia, Universitá di Firenze, via La Pira 4, 50121 Firenze, Italy; b Dipartimento di Scienze della Terra, Universitá di Firenze, via La Pira 4, 50121 Firenze, Italy

## Abstract

A structural refinement of a natural sample of a Co-bearing mansfieldite, AlAsO_4_·2H_2_O [aluminium orthoarsenate(V) dihydrate], has been performed based on synchrotron powder diffraction data, with 5% of the octa­hedral Al sites replaced by Co. Mansfieldite is the aluminium analogue and an isotype of the mineral scorodite (FeAsO_4_·2H_2_O), with which it forms a solid solution. The framework structure is based on AsO_4_ tetra­hedra sharing their vertices with AlO_4_(H_2_O)_2_ octa­hedra. Three of the four H atoms belonging to the two water mol­ecules in *cis* positions take part in O—H⋯O hydrogen bonding.

## Related literature

Mansfieldite (AlAsO_4_·2H_2_O) was first described by Allen *et al.* (1948 ▶) and the synthetic analogue was structurally charac­terised by Harrison (2000 ▶). For the structures of isotypic minerals and synthetic compounds, see: Botelho *et al.* (1994 ▶), Tang *et al.* (2001 ▶) (yanomamite, InAsO_4_·2H_2_O); Kniep *et al.* (1977 ▶) (variscite, AlPO_4_·2H_2_O); Hawthorne (1976 ▶), Kitahama *et al.* (1975 ▶), Xu *et al.* (2007 ▶) (scorodite, FeAsO_4_·2H_2_O); Taxer & Bartl (2004 ▶) (strengite, FePO_4_·2H_2_O); Loiseau *et al.* (1998 ▶) (synthetic GaPO_4_·2H_2_O); Mooney-Slater (1961 ▶) (synthetic InPO_4_·2H_2_O and TlPO_4_·2H_2_O).

## Experimental

### 

#### Crystal data

AlAsO_4_·2H_2_O
*M* *_r_* = 203.53
Orthorhombic, 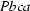

*a* = 8.79263 (11) Å
*b* = 9.79795 (10) Å
*c* = 10.08393 (11) Å
*V* = 868.73 (2) Å^3^
*Z* = 8
Synchrotron radiation
λ = 0.68780 Å
μ = 7.25 mm^−1^
*T* = 298 K
Specimen shape: flat sheet
5.0 × 5.0 × 0.4 mm
Particle morphology: powder, light pink

#### Data collection

ESRF BM08 beamline
Specimen mounted in transmission mode
Scan method: fixed
Absorption correction: for a cylinder mounted on the ϕ axis *T*_min_ = 0.072, *T*_max_ = 0.095
2θ_min_ = 6.0, 2θ_max_ = 53.0°
Increment in 2θ = 0.01°

#### Refinement

*R*_p_ = 0.039
*R*_wp_ = 0.050
*R*_exp_ = 0.039
*R*_B_ = 0.034
*S* = 1.31
Excluded region(s): none
Profile function: CW pseudo-Voigt
60 parameters
No restraints
H-atom parameters not refined
Preferred orientation correction: Spherical harmonics ODF

### 

Data collection: local image plate reading software; cell refinement: *GSAS* (Larson & Von Dreele, 2004 ▶) and *EXPGUI* (Toby, 2001 ▶); data reduction: *FIT2D* (Hammersley, 1997 ▶); program(s) used to solve structure: atomic coordinates from Harrison (2000 ▶); program(s) used to refine structure: *GSAS* and *EXPGUI*; molecular graphics: *VICS* (Izumi & Dilanian, 2005 ▶); software used to prepare material for publication: *publCIF* (Westrip, 2009 ▶).

## Supplementary Material

Crystal structure: contains datablocks global, I. DOI: 10.1107/S1600536808044127/wm2209sup1.cif

Rietveld powder data: contains datablocks I. DOI: 10.1107/S1600536808044127/wm2209Isup2.rtv

Additional supplementary materials:  crystallographic information; 3D view; checkCIF report

## Figures and Tables

**Table 1:** Selected bond lengths (Å)

Al1—O1	1.908 (4)
Al1—O2^i^	1.906 (4)
Al1—O3^ii^	1.850 (4)
Al1—O4^iii^	1.848 (4)
Al1—O5*w*	1.914 (4)
Al1—O6*w*	1.988 (4)
As2—O1	1.676 (3)
As2—O2	1.654 (3)
As2—O3	1.704 (4)
As2—O4	1.682 (4)

Symmetry codes: (i) 
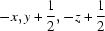
; (ii) 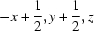; (iii) 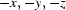.

**Table 2:** Hydrogen-bond geometry (Å, °)

*D*—H⋯*A*	*D*—H	H⋯*A*	*D*⋯*A*	*D*—H⋯*A*
O5*w*—H51⋯O4^i^	0.83	1.78	2.607 (5)	168 (1)
O5*w*—H52⋯O1^iv^	0.71	2.03	2.614 (5)	161 (1)
O6*w*—H62⋯O3	0.93	1.71	2.587 (5)	156 (1)

Symmetry codes: (i) 
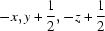
; (iv) 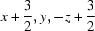.

**Fig. 1.**
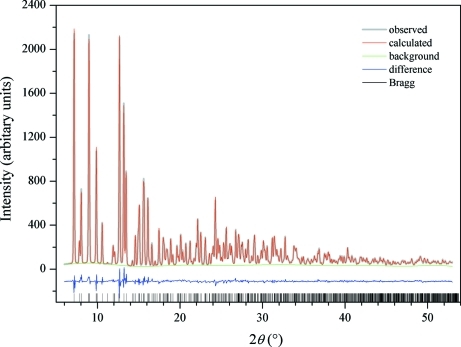
The observed, calculated, background and difference X-ray diffraction profile for natural mansfieldite. Bragg reflection positions are shown at the bottom.

**Fig. 2.**
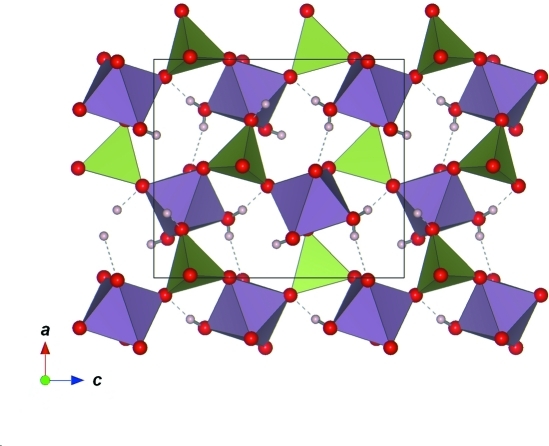
The crystal structure of mansfieldite, viewed along the b axis. The unit cell is outlined and the hydrogen bonds are represented by dashed lines.

## supplementary crystallographic information

### Comment

The mineral mansfieldite belongs to the general group of hydrous arsenates, a structure subgroup of variscite (AlPO_4_.2H_2_O), and has been known since the discovery of Allen *et al.* (1948). It is of white to pale gray colour and has a vitreous luster. It often develops encrustations, crust-like or rounded aggregates on the matrix, and individual crystals are rarely observed. While the structural data of synthetic mansfieldite were reported recently by Harrison (2000), no data regarding natural samples have been provided in literature up to now, probably because of the rare occurrence of crystalline material suitable for structural investigations. Rietveld refinement of a natural sample of a Co-bearing mansfieldite has now been carried out using synchrotron powder diffraction data (Fig. 1).

Mansfieldite crystallizes in the *Pbca* space group and is isostructural with the the arsenate minerals scorodite [FeAsO_4_.2H_2_O] (Xu *et al.*, 2007; Hawthorne, 1976; Kitahama *et al.*, 1975), yanomamite [InAsO_4_.2H_2_O] (Tang *et al.*, 2001; Botelho *et al.*, 1994), TlAsO_4_.2H_2_O (Mooney-Slater, 1961), and the phosphate minerals variscite [AlPO_4_.2H_2_O] (Kniep *et al.*, 1977), strengite [FePO_4_.2H_2_O] (Taxer & Bartl, 2004), InPO_4_.2H_2_O (Tang *et al.*, 2001; Mooney-Slater, 1961), GaPO_4_.2H_2_O (Loiseau *et al.*, 1998) and TlPO_4_.2H_2_O (Mooney-Slater, 1961). Correspondingly to the mentioned arsenates, the structure of mansfieldite is composed of AsO_4_ tetrahedra and AlO_4_(H_2_O)_2_ octahedra, each tetrahedron being connected to four octahedra and each octahedron being connected to four tetrahedra, as shown in Fig. 2. The interatomic distances Al—O and As—O resulting from the refinement of the structure are reported in Table 1. The two water molecules are located in *cis* position, but the O atoms O5W and O6W do not participate in the linkage with the As—O_4_ tetrahedra, while three of the hydrogen atoms (H1, H2 and H3) take part in D—H···A bonds that link O5W to O4, O5W to O1, and O6W to O3, respectively (Table 2). Such a structure framework displays channels along *b*. With respect to the synthetic mansfieldite (Harrison, 2000), the natural sample shows a slightly smaller unit cell volume, which is the effect of slightly smaller octahedral (9.110 *versus* 9.187 Å^3^) and tetrahedral (2.428 *versus* 2.450 Å^3^) volumes. Also the distortion index of both the polyhedra, 0.019 and 0.008 for the octahedral and tetrahedral site, respectively, is larger than that calculated for the synthetic material, *viz.* 0.014 and 0.002. A slight distortion of the structure is probably due to the small amount of incorporated Co and other elements present in the structure of the natural sample. Bond valence calculations show slightly overbonded values of 3.036 and 5.079 valence units for the cation in the octahedral site and in the tetrahedral site, respectively. The isotropic thermal parameters for O5W and O6W, 0.0182 (12) and 0.0164 (12) Å^2^, respectively, are slightly larger than those of the other oxygen atoms and reveal a certain degree of disorder of the water molecules along the channels, since they are not taking part in the metal-oxygen-metal chains of the structure.

### Experimental

The specimen used in this study is from the locality of Mt. Cobalt, Cloncurry District, Queensland, Australia. Preliminarily, some transparent single crystals were selected from massive, light purple coloured, mansfieldite associated with smolianinovite [(Co,Ni,Mg,Ca)_3_(Fe^3+^,Al)_2_(AsO_4_)_4_.11H_2_O]. The average elemental chemical composition, determined using electron microprobe analyses, yielded the empirical chemical formula, calculated on a total of two cations per formula unit, (Al_0.944_Co^3+^_0.046_Cu^2+^_0.005_ Fe^3+^_0.003_ Zn^2+^_0.002_)_Σ__=1_ (As_0.972_Al_0.022_P_0.006_)_Σ__=1_O_3.975_.2H_2_O resulting in the simplified formula AlAsO_4_.2H_2_O. The excess Al resulting from the calculation has been arbitrarily assigned to the tetrahedral site. X-ray data collections of some single crystals, with a CCD equipped diffractometer, revealed that all the samples were actually polycrystalline aggregates and showed irregular and broadened spots typical of materials with high mosaicity. Refinements from single-crystal X-ray diffraction data yielded, in the best case, a not satisfactorily *R*_F_ index of 6.54%. Fragments of pure mansfieldite were then ground and used for synchrotron X-ray data collection.

### Refinement

Structural data were refined employing the Rietveld method and starting from the atomic coordinates provided by Harrison (2000), except for the H atom parameters that were not refined but included in the model. The site occupancies were assigned according to the composition of the idealised chemical formula (Al_0.95_Co^3+^_0.05_)AsO_4_.2H_2_O, with 5% Co at the octahedral Al sites.

### Crystal data

**Table:**

AlAsO_4_·2H_2_O	*F*(000) = 789
*M**_r_* = 203.53	*D*_x_ = 3.112 Mg m^−^^3^
Orthorhombic, *P**b**c**a*	Synchrotron radiation, λ = 0.68780 Å
Hall symbol: -P 2ac 2ab	µ = 7.25 mm^−^^1^
*a* = 8.79263 (11) Å	*T* = 298 K
*b* = 9.79795 (10) Å	Particle morphology: powder
*c* = 10.08393 (11) Å	light pink
*V* = 868.73 (2) Å^3^	flat sheet, 5.0 × 5.0 mm
*Z* = 8	

### Data collection

**Table:**

ESRF BM08 Beamline diffractometer	Absorption correction: for a cylinder mounted on the φ axis Debye-Scherrer, Term (= MU.r/wave) = 2.4540. Correction is not refined.
Data collection mode: transmission	*T*_min_ = 0.072, *T*_max_ = 0.095
Scan method: Stationary detector	

### Refinement

**Table:**

Refinement on *I*_net_	Excluded region(s): none
Least-squares matrix: full	Profile function: CW Pseudo-Voigt
*R*_p_ = 0.039	60 parameters
*R*_wp_ = 0.050	no restraints
*R*_exp_ = 0.039	H-atom parameters not refined
*R*_Bragg_ = 0.034	*w* = 1/[Y_i_]
*R*(*F*^2^) = 0.03400	(Δ/σ)_max_ = 0.01
χ^2^ = 1.716	Background function: GSAS Background function number 1 with 14 terms. Shifted Chebyshev function of 1st kind
? data points	Preferred orientation correction: Spherical Harmonics ODF

### Fractional atomic coordinates and isotropic or equivalent isotropic displacement parameters (Å^2^)

**Table:**

	*x*	*y*	*z*	*U*_iso_*/*U*_eq_	Occ. (<1)
Al1	0.1478 (3)	0.18068 (16)	0.12682 (18)	0.0087 (5)*	0.95
Co1	0.1478 (3)	0.18068 (16)	0.12682 (18)	0.0087 (5)*	0.05
As2	0.03632 (9)	−0.13857 (6)	0.15042 (6)	0.00875 (18)*	
O1	0.0106 (4)	0.0308 (3)	0.1440 (3)	0.0055 (8)*	
O2	0.0003 (5)	−0.1986 (3)	0.3005 (3)	0.0077 (12)*	
O3	0.2208 (5)	−0.1729 (4)	0.1105 (3)	0.0086 (12)*	
O4	−0.0815 (5)	−0.2171 (3)	0.0434 (4)	0.0063 (11)*	
O5w	0.2296 (5)	0.1244 (4)	0.2939 (3)	0.0182 (12)*	
O6w	0.3186 (4)	0.0696 (4)	0.0559 (3)	0.0164 (12)*	
H51	0.19	0.17	0.354	0.08*	
H52	0.309	0.114	0.301	0.036*	
H61	0.347	0.093	−0.01	0.033*	
H62	0.295	−0.023	0.053	0.047*	

### Geometric parameters (Å, °)

**Table:**

Al1—O1	1.9080 (35)	As2—O4	1.682 (4)
Al1—O2^i^	1.906 (4)	O5W—H51	0.8295
Al1—O3^ii^	1.850 (4)	O5W—H52	0.709
Al1—O4^iii^	1.848 (4)	O6W—H61	0.746
Al1—O5w	1.914 (4)	O6W—H62	0.931
Al1—O6w	1.988 (4)	O1—H52^iv^	2.028
As2—O1	1.6760 (29)	O3—H62	1.709
As2—O2	1.6539 (33)	O4—H51^v^	1.790
As2—O3	1.704 (4)		
			
O1—Al1—O2^vi^	90.64 (19)	O1—As2—O3	108.35 (18)
O1—Al1—O3^ii^	179.4743 (18)	O1—As2—O4	110.18 (18)
O1—Al1—O4^iii^	91.93 (18)	O2—As2—O3	109.15 (21)
O1—Al1—O5w	86.31 (15)	O2—As2—O4	107.83 (19)
O1—Al1—O6w	95.09 (18)	O3—As2—O4	110.13 (18)
O2^vi^—Al1—O3^ii^	88.84 (18)	Al1—O1—As2	132.87 (23)
O2^vi^—Al1—O4^iii^	91.28 (18)	Al1^vii^—O2—As2	134.72 (23)
O2^vi^—Al1—O5w	95.56 (17)	Al1^viii^—O3—As2	136.63 (23)
O2^vi^—Al1—O6w	173.93 (23)	Al1^iii^—O4—As2	134.59 (22)
O3^ii^—Al1—O4^iii^	87.99 (19)	Al1—O5w—H51	109.26 (34)
O3^ii^—Al1—O5w	93.84 (18)	Al1—O5w—H52	120.00 (32)
O3^ii^—Al1—O6w	85.43 (20)	H51—O5w—H52	114.6
O4^iii^—Al1—O5w	172.94 (19)	Al1—O6w—H61	113.93 (35)
O4^iii^—Al1—O6w	90.54 (17)	Al1—O6w—H62	112.12 (32)
O5w—Al1—O6w	82.82 (17)	H61—O6w—H62	110.3
O1—As2—O2	111.20 (17)		

Symmetry codes:  (i) −*x*, *y*+1/2, −*z*+1/2; (ii) −*x*+1/2, *y*+1/2, *z*; (iii) −*x*, −*y*, −*z*; (iv) *x*−1/2, *y*, −*z*+1/2; (v) −*x*, *y*−1/2, −*z*+1/2; (vi) −*x*, *y*+3/2, −*z*+3/2; (vii) −*x*−1, *y*+1/2, −*z*+1/2; (viii) −*x*+1/2, *y*−1/2, *z*.

### Hydrogen-bond geometry (Å, °)

**Table:**

*D*—H···*A*	*D*—H	H···*A*	*D*···*A*	*D*—H···*A*
O5w—H51···O4^i^	0.8295	1.784	2.607 (5)	168.19 (28)
O5w—H52···O1^ix^	0.709	2.028	2.614 (5)	160.98 (27)
O6w—H62···O3	0.931	1.709	2.587 (5)	155.80 (26)

Symmetry codes: (i) −*x*, *y*+1/2, −*z*+1/2; (ix) *x*+3/2, *y*, −*z*+3/2.

## References

[bb1] Allen, V. T., Fahey, J. J. & Axelrod, J. M. (1948). *Am. Mineral.* **33**, 122–134.

[bb2] Botelho, N. F., Roger, G., d’Yvoire, F., Moëlo, Y. & Volfinger, M. (1994). *Eur. J. Mineral.* **6**, 245–254.

[bb3] Hammersley, A. P. (1997). *FIT2D* Internal Report No. ESRF97HA02T. ESRF, Grenoble, France.

[bb4] Harrison, W. T. A. (2000). *Acta Cryst.* C**56**, e421.

[bb5] Hawthorne, F. C. (1976). *Acta Cryst.* B**32**, 2891–2892.

[bb6] Izumi, F. & Dilanian, R. A. (2005). *IUCr Commission on Powder Diffraction Newsletter*, No. 32, pp. 59–63.

[bb7] Kitahama, K., Kiriyama, R. & Baba, Y. (1975). *Acta Cryst.* B**31**, 322–324.

[bb8] Kniep, R., Mootz, D. & Vegas, A. (1977). *Acta Cryst.* B**33**, 263–265.

[bb9] Larson, A. C. & Von Dreele, R. B. (2004). *GSAS* Report No. LAUR86-748. Los Alamos National Laboratory, New Mexico, USA.

[bb10] Loiseau, T., Paulet, C. & Ferey, G. (1998). *C. R. Acad. Sci. Ser. IIc Chim.* **1**, 667–674.

[bb11] Mooney-Slater, R. C. L. (1961). *Acta Cryst.* **14**, 1140–1146.

[bb12] Tang, X., Gentiletti, M. J. & Lachgar, A. (2001). *J. Chem. Crystallogr.* **31**, 45–50.

[bb13] Taxer, K. & Bartl, H. (2004). *Cryst. Res. Technol.* **39**, 1080–1088.

[bb14] Toby, B. H. (2001). *J. Appl. Cryst.* **34**, 210–213.

[bb15] Westrip, S. P. (2009). *publCIF* In preparation.

[bb16] Xu, Y., Zhou, G.-P. & Zheng, X.-F. (2007). *Acta Cryst.* E**63**, i67–i69.

